# Disability among Palestinian patients with inflammatory bowel disease: evaluation using the IBD Disk and identification of associated factors

**DOI:** 10.1093/crocol/otag079

**Published:** 2026-07-17

**Authors:** Ahmed Quraish, Ro’ya Khanfar, Karmil Kharouf, Sami Salous, Dala Quraish, Qusay Abdoh

**Affiliations:** Department of Medicine, Faculty of Medicine and Allied Medical Sciences, An-Najah National University, Nablus 44839, Palestine; Department of Scientific Research and Medical Projects, Faculty of Medicine and Allied Medical Sciences, An-Najah National University, Nablus 44839, Palestine; Department of Medicine, Faculty of Medicine and Allied Medical Sciences, An-Najah National University, Nablus 44839, Palestine; Department of Medicine, Faculty of Medicine and Allied Medical Sciences, An-Najah National University, Nablus 44839, Palestine; Department of Medicine, Faculty of Medicine and Allied Medical Sciences, An-Najah National University, Nablus 44839, Palestine; Department of Medicine, Faculty of Medicine and Allied Medical Sciences, An-Najah National University, Nablus 44839, Palestine; Department of Medicine, Faculty of Medicine and Allied Medical Sciences, An-Najah National University, Nablus 44839, Palestine; Gastrointestinal Medical Center, Nablus, 4050086, Palestine

**Keywords:** inflammatory bowel disease, disability, IBD Disk, Crohn’s disease, ulcerative colitis

## Abstract

**Background:**

Inflammatory bowel disease (IBD) can substantially impair daily functioning, yet disability prevalence and determinants remain underexplored in Palestine. This study aimed to assess the prevalence of IBD-related disability using the IBD Disk tool and to identify risk factors associated with high disability among Palestinian patients.

**Methods:**

A cross-sectional study was conducted among patients with a confirmed diagnosis of Crohn’s disease (CD) or ulcerative colitis for at least six months. Participants completed an Arabic translation of the IBD Disk prepared through forward-backward translation. Demographic, clinical, and lifestyle data were collected. Disability was defined as an IBD Disk total score >40. Logistic regression was used to identify factors independently associated with disability.

**Results:**

A total of 179 patients (59.2% CD, mean age 33.68 ± 12.27 years) participated. The prevalence of IBD-related disability was 58.1%, with a mean total IBD Disk score of 45.31 ± 23.20. Joint pain was the highest-scoring domain (6.06 ± 3.32), while sexual function scored lowest (2.46 ± 2.43). While azathioprine use (adjusted odds ratio [aOR] 0.421, 95% confidence interval [CI] 0.213-0.829, *P* = .012) was associated with lower disability, self-reported vitamin D deficiency in the preceding 3 months was associated with higher disability (aOR 2.932, 95% CI 1.066-8.060, *P* = .03). Among patients with CD, cigarettes smoked per day showed a weak positive correlation with IBD Disk scores.

**Conclusions:**

High disability was common among Palestinian patients with IBD. Self-reported vitamin D deficiency in the preceding 3 months was associated with higher odds of high disability, whereas azathioprine use was associated with lower odds. Cigarettes smoked per day showed a weak positive correlation with disability scores among patients with CD, but smoking status was not independently associated with high disability.

## Graphical abstract

**Graphical Abstract:**
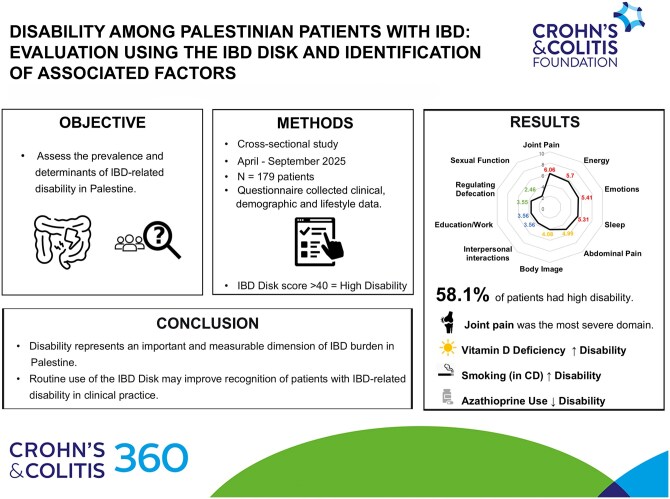

## Introduction

Inflammatory bowel disease (IBD), including Crohn’s disease (CD) and ulcerative colitis (UC), is a chronic, relapsing-remitting disease characterized by alternating active and remission episodes of intestinal inflammation.^1^ A complex interplay of host genetic, microbial, and environmental variables contributes to its pathogenesis. However, there are still unanswered questions regarding the etiology and behavior of the disease.^2^ Globally, the burden of IBD is a significant public health challenge. This burden is mainly due to the increased prevalence, especially in newly industrialized areas in South America, Asia, and Africa, which places an increasing social and financial strain on society, governments, and healthcare systems.^3–5^ Compared with the general population, patients with IBD experience reduced quality of life and higher rates of disability.^6–8^

Even though IBD is linked to substantial morbidity and disability, which at times necessitates hospitalization and surgery, physicians frequently underestimate the impact of this disease on their patients’ lives.^9^^,^^10^ IBD-related disability occurs due to several factors, with the nature of it being an incurable relapsing-remitting disease with heterogeneous clinical presentation being a major one. Another factor is its association with extraintestinal systemic symptoms such as fatigue and occasionally fever besides cutaneous, hepatobiliary, rheumatological, and ophthalmological manifestations.^11–13^ Physical, psychological, familial, and social aspects of this illness also play a role in disability.^14^^,^^15^

In recent years, preventing disability has garnered increasing attention as there has been a significant change in the treatment paradigm from symptom control to complete disease control (clinical and endoscopic relief). The end goal of such change has been to stop the disease’s progression and preventing intestinal damage and subsequently the ensuing disability.^16^ The updated Selecting Therapeutic Targets in Inflammatory Bowel Disease further highlights that in addition to clinical remission and endoscopic healing, major long-term goals focus on the absence of disability, restoration of quality of life, and normal growth in children.^17^

Adopting the World Health Organization (WHO) definition, IBD-related disability includes restrictions on one’s ability to pursue education and employment, exclusion from social and economic activities, and impairments of one’s physical and psychological functioning.^18^ In an effort to measure disability, the IBD Disability Index (IBD-DI) was developed in collaboration with the WHO.^19^ It allows patients to be followed to evaluate the effects of treatment plans and pathways on the progression of illnesses, the degree of disability, and the overall impact of the condition.^10^ While the IBD-DI is a useful tool for assessing disability, its length often limits its use in busy clinical settings. Recently, a shortened self-administered tool derived from the IBD-DI, called the IBD Disk, was developed. This tool was created for routine clinical assessment as a more convenient way of capturing disability caused by IBD.^20^ Despite the increasing burden of IBD in many regions, including the Middle East, IBD-related disability remains an understudied issue. To our knowledge, there has been no study assessing IBD disability using the IBD Disk in Palestine. Therefore, this study aimed to determine the prevalence of IBD-related disability among Palestinian patients using the IBD Disk and to identify the risk factors associated with higher disability.

## Materials and methods

### Study design and setting

A cross-sectional study was conducted in the Northern West Bank, Palestine from April 2025 to September 2025. The study assessed IBD-related disability and associated risk factors among patients with IBD attending follow-up visits at multiple general outpatient gastroenterology clinics across the Northern West Bank. Recruitment was clinic-based and involved patients receiving follow-up care in general gastroenterology practice rather than in specialized IBD referral clinics or through a centralized registry or database.

### Participants and sampling

A convenience sampling method was employed, where all those attending outpatient follow-up visits who met the eligibility criteria were invited to participate in the study. Participants included in the study were those with a confirmed diagnosis of CD or UC established by a gastroenterologist based on endoscopic and histologic evaluation. Inclusion criteria also included disease duration ≥6 months. All ages were included provided that they were able to independently complete the Arabic questionnaire. Patients who had a diagnosis other than IBD, filled incomplete questionnaires, or were unable to complete the questionnaire due to cognitive or language limitations, were excluded from the study. Because recruitment was based on convenience sampling during clinic attendance and the number of eligible patients approached was not systematically recorded, a formal survey response rate could not be calculated. Recruitment was limited to patients attending outpatient follow-up visits during the study period.

The sample size for this study was calculated using the Raosoft sample size calculator. Based on an estimated population of 367 patients, assuming a 95% confidence level, a 5% margin of error, and an expected outcome prevalence of 19% derived from a Saudi IBD Disk-based study, the minimum required sample size was 145 patients.^21^^,^^22^ The final sample size collected was 179, exceeding the minimum required sample size.

### Data collection and study variables

Data were collected using a structured Arabic questionnaire distributed as a Google Form. Questionnaires were completed by participants during clinic visits with trained researchers available to clarify any items. All variables were obtained through patient self-report, including height and weight, from which body mass index (BMI) was calculated. The questionnaire was made up of several sections covering sociodemographic characteristics, lifestyle factors, anthropometric information, disease-related variables, treatment history, comorbidities, micronutrient deficiencies, and the IBD Disk instrument. All reported clinical characteristics, comorbidities, treatment exposures, smoking variables, and hospitalization or surgical history were based on participant self-report and were not independently verified using medical records, laboratory data, or endoscopic/biochemical assessments. Self-reported micronutrient deficiencies referred to the preceding 3 months. Current supplement and medication use was recorded separately. Accordingly, objective disease activity indices such as clinical scores, inflammatory biomarkers, or endoscopic severity measures were not available for analysis.

Collected variables included demographic and clinical characteristics including age, gender, marital status, education level, BMI, smoking status (cigarettes), number of cigarettes per day, waterpipe/vape use, disease type (CD vs UC), disease duration, history of surgery, history of hospitalization, adherence to treatment, adherence to follow-up visits, use of 5-aminosalicylates (5-ASA), use of azathioprine, use of biologic therapy, use of systemic corticosteroids, use of other medications, presence of other chronic diseases (diabetes mellitus, hypertension, dyslipidemia, Behçet’s disease, rheumatoid arthritis, psoriasis, osteoporosis, and others), and presence of any self-reported nutrient deficiency in the preceding 3 months (vitamin D deficiency, iron deficiency, vitamin B12 deficiency, calcium deficiency, and other deficiencies). The primary outcome was IBD-related disability, measured using the total IBD Disk score (0–100). For analytic purposes, participants were categorized as having high disability (IBD Disk score >40) or lower disability burden (IBD Disk score ≤40).

### IBD Disk instrument

The IBD Disk is made up of 10 domains. The components include abdominal pain, regulating defecation, interpersonal interactions, education and work, sleep, energy, emotions, body image, sexual functions, and joint pain. Sub-scores for each of the ten components in the IBD Disk questionnaire provide information regarding the patient’s impairment, systemic symptoms, manifestations, and overall effects on social life. Responses are provided on a scale ranging from 0 to 10 (0 = no burden; 10 = maximal burden). The total score was out of 100; the best score is 0, while the worst score is 100. A cutoff score of >40 was used to determine high disability as it has been linked to a high daily-life burden from IBD.^23^

The original English IBD Disk questionnaire was translated into Arabic using a forward-backward translation process conducted by independent bilingual translators. The final translated version was then reviewed and approved by a gastroenterologist to confirm its clinical relevance and conceptual accuracy. Prior to data collection, the translated questionnaire was pilot tested with 10 patients with IBD to assess item clarity, comprehension, wording, and feasibility of completion in the target patient population. A small sample of 10 patients with IBD was considered appropriate for this preliminary linguistic and face-validity pretest because the aim was to identify major comprehension or wording problems before full data collection, rather than to perform formal psychometric validation. As no major comprehension difficulties were identified and only minor wording revisions were required, the pilot sample was not expanded before full recruitment. Internal consistency was subsequently assessed in the full study sample using Cronbach’s alpha, which yielded a value of 0.89.

### Statistical analysis

For data analysis, we used IBM Statistical Package for the Social Sciences. Descriptive statistics were calculated and presented as frequencies and percentages, while continuous variables were summarized as means ± standard deviations. Normality of continuous variables was assessed using the Shapiro–Wilk test. Because total IBD Disk score and individual domain scores were not normally distributed, comparisons between patients with CD and UC were performed using the Mann–Whitney *U* test. Pearson’s chi-square test and Fisher’s exact test (when expected cell counts were less than 5) were used to determine the associations between various predictor variables and outcome (high disability vs lower disability burden). Independent predictors of disability were identified using multivariable binary logistic regression with results reported as adjusted odds ratios (aORs) with 95% confidence intervals (CIs). Variables with a *P*-value < .20 in the univariate analysis, as well as clinically relevant factors identified, were entered into the multivariable binary logistic regression model. Spearman’s rank correlation was used to assess associations between the continuous IBD Disk score and continuous variables. Exploratory subgroup analysis was additionally performed by stratifying patients according to biologic therapy status and prior surgical history. Differences in total IBD Disk score and individual domains were also assessed using the Mann–Whitney *U* test. A *P*-value less than .05 was considered significant.

### Ethical considerations

IRB (Institutional Review Board) approval was obtained prior to questionnaire distribution [Reference: Med. March. 2025/36]. Written informed consent was obtained from all adult participants prior to participation. For participants aged below 18 years, written informed consent was obtained from a parent or legal guardian, and participant assent was obtained when applicable. Confidentiality, anonymity, and voluntary participation were ensured.

## Results

### Characteristics of the patients

A total of 179 patients with IBD were included in this study. The mean age was 33.68 ± 12.27 and 93 (52.0%) were male. Most patients, 111 (62.0%) were married and 102 (57.0%) had university degrees. As for disease distribution, the majority of patients, 106 (59.2%) had CD, while 73 (40.8%) were diagnosed with UC. The mean duration of the disease was 8.43 ± 6.68 years.

In terms of lifestyle factors, 66 (36.9%) were cigarette smokers with a mean of 4.29 ± 10.17 cigarettes per day. Nearly half of the patients, 88 (49.4%) had a normal BMI while 78 (43.8%) were overweight or obese. The mean BMI was 26.08 ± 4.73 kg/m^2^. Comorbidities reported by patients were relatively infrequent. Hypertension 10 (5.6%) and rheumatoid arthritis 8 (4.5%) were the most common. Other comorbidities included diabetes mellitus 7 (3.9%), dyslipidemia 5 (2.8%), psoriasis 3 (1.7%), osteoporosis 2 (1.1%), and Behçet’s disease 2 (1.1%).

Regarding treatment, 58 (32.4%) were using 5-aminosalicylates, 68 (38.0%) were using azathioprine, 48 (26.8%) were on biologic treatment, and 19 (10.6%) were taking systemic corticosteroids. Self-reported medication and follow-up adherence was high, with (87.7%) reporting commitment to treatment and 127 (70.9%) committed to regular follow-ups.

More than half of the patients, 115 (64.2%) had a history of IBD-related hospitalization. A total of 31 (17.3%) patients reported having IBD-related surgery, with 19 (61.3%) of them undergoing bowel resections. More details are seen in Table 1.

**Table 1:** Demographic and clinical characteristics of the study participants (*N* = 179).

Variable	Total: *n* (%) or mean ± SD
**Age, years (mean ± SD)**	33.68 ± 12.27
**Age groups**	
** 18 and younger**	9 (5.0)
** 19-30**	71 (39.7)
** 31-50**	78 (43.6)
** 51 and older**	21 (11.7)
**Gender**	
** Male**	93 (52.0)
** Female**	86 (48.0)
**Marital status**	
** Single**	66 (36.9)
** Married**	111 (62.0)
** Divorced**	1 (0.6)
** Widow**	1 (0.6)
**Years of marriage, years (mean ± SD)**	15.33 ± 11.42
**Education**	
** Primary school**	8 (4.5)
** High school**	61 (34.1)
** University**	102 (57.0)
** Postgraduate**	8 (4.5)
**Disease type**	
** Crohn’s disease**	106 (59.2)
** Ulcerative colitis**	73 (40.8)
**Disease duration, years (mean ± SD)**	8.43 ± 6.68
**Disease duration**	
** Less than 5 years**	53 (29.6)
** 5-10 years**	82 (45.8)
** more than 10 years**	44 (24.6)
**Committed to treatment**	
** Yes**	157 (87.7)
** No**	22 (12.3)
**Committed to regular follow-ups**	
** Yes**	127 (70.9)
** No**	52 (29.1)
**Reason for not being committed to doctor visit**	
** Stable**	26 (14.5)
** Medications not available**	1 (0.6)
** Difficult transportation**	9 (5)
** Not committed**	6 (3.4)
** Financial**	7 (3.9)
**Medication**	
** 5-aminosalicylates**	58 (32.4)
** Azathioprine**	68 (38.0)
** Biologic**	48 (26.8)
** Systemic corticosteroids**	19 (10.6)
** Other medications**	34 (19.6)
**Hospitalization**	
** No**	64 (35.7)
** Yes**	115 (64.2)
**Had surgery**	
** No**	148 (82.7)
** Yes**	31 (17.3)
**Type of surgery**	
** Bowel resection**	19 (10.6)
** Hemorrhoid/Fistula**	5 (2.8)
** Other abdominal surgery (appendix, cholecystectomy)**	7 (3.9)
**Other chronic diseases**	
** Diabetes Mellitus**	7 (3.9)
** Hypertension**	10 (5.6)
** Dyslipidemia**	5 (2.8)
** Behçet’s disease**	2 (1.1)
** Rheumatoid arthritis**	8 (4.5)
** Psoriasis**	3 (1.7)
** Osteoporosis**	2 (1.1)
** Others**	15 (8.4)
**BMI**	
** Underweight**	12 (6.7)
** Normal**	88 (49.4)
** Overweight**	46 (25.8)
** Obese**	32 (18)
**Cigarette smoker**	
** No**	113 (63.1)
** Yes**	66 (36.9)
**Cigarettes per day, number/day (mean ± SD)**	4.29 ± 10.17
**Smoker (other than cigarette)**	
** Nothing**	149 (83.0)
** Waterpipe**	27 (15.1)
** Vape**	3 (1.7)

Abbreviations: BMI, body mass index; SD, standard deviation.

### IBD-related disability scores

**Figure 1:**
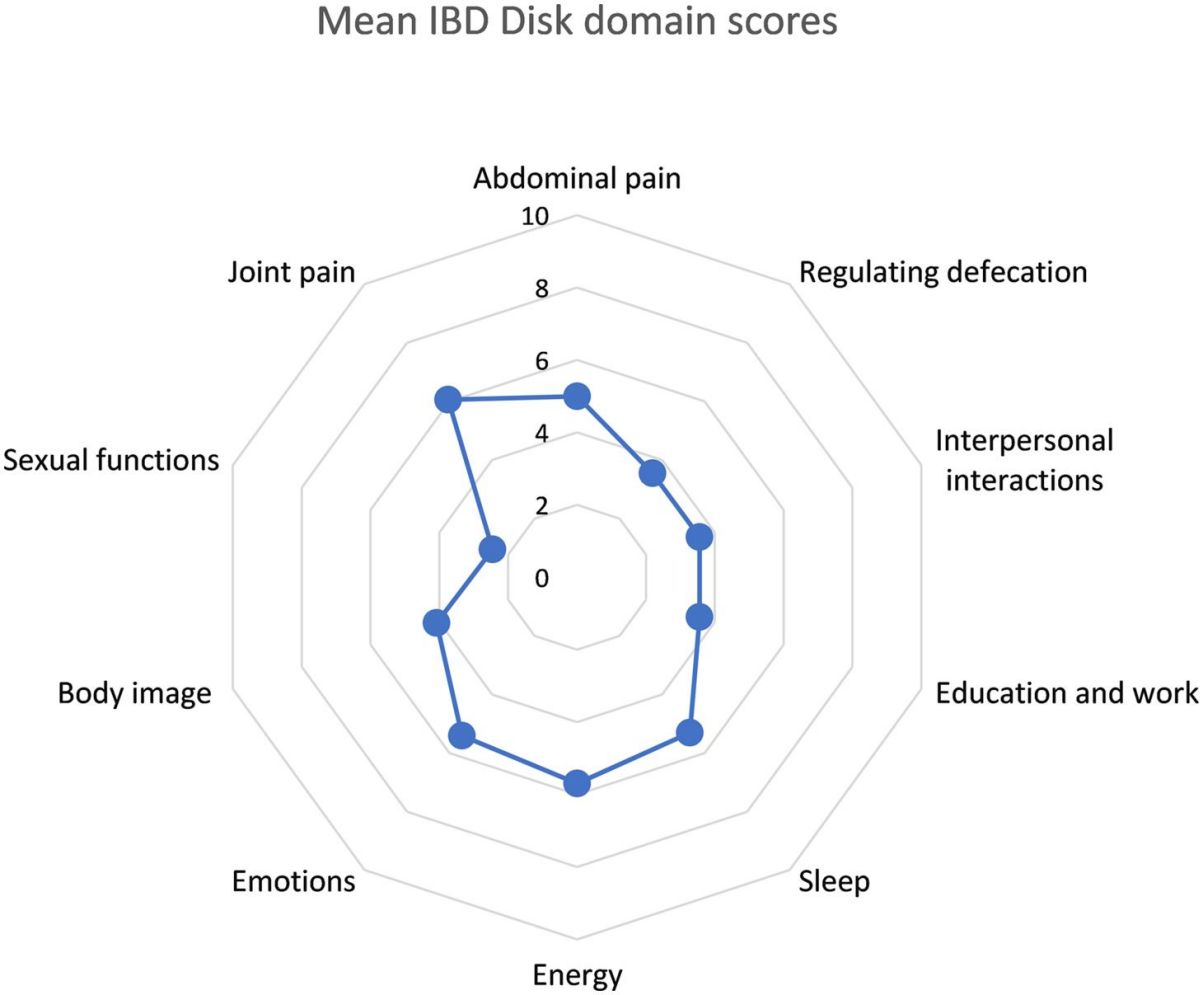
Radar plot showing mean IBD Disk domain scores among Palestinian patients with inflammatory bowel disease. Higher scores indicate greater impairment in the corresponding domain (scale 0-10). IBD, inflammatory bowel disease.

The mean total IBD Disk score in this study was 45.31 ± 23.2. Using a cutoff value of more than 40 to define high disability, 104 (58.1%) patients were classified as having high disability, whereas 75 (41.9%) were classified as having lower disability burden (IBD Disk score ≤40). The highest mean scores observed across the 10 IBD Disk domains were joint pain with a mean of 6.06 ± 3.32, tiredness/lack of energy (5.70 ± 3.33), emotional wellbeing (5.41 ± 3.49), and sleep disturbances (5.31 ± 3.46). The lowest score was observed in the sexual domain (2.46 ± 2.43). A visual representation of the overall disability profile is provided in Figure 1, which demonstrates the distribution of mean domain scores across the IBD Disk. Mean total IBD Disk scores were similar between patients with CD and those with UC (45.92 ± 23.07 vs 44.42 ± 23.52, *P* = .564). No significant differences were observed between disease types across any individual IBD Disk domain scores (Table 2).

**Table 2:** IBD Disk total and domain scores overall and by disease type.

Variable	Total, mean ± SD	UC, mean ± SD	CD, mean ± SD	*P*-value
**Total IBD Disk score**	45.31 ± 23.20	44.42 ± 23.52	45.92 ± 23.07	.564
**Abdominal pain**	4.99 ± 3.16	4.52 ± 2.95	5.31 ± 3.26	.086
**Regulating defecation**	3.55 ± 3.05	3.51 ± 3.07	3.58 ± 3.06	.736
**Interpersonal interactions**	3.56 ± 3.17	3.93 ± 3.27	3.30 ± 3.09	.172
**Education and work**	4.18 ± 3.43	4.15 ± 3.33	4.21 ± 3.51	.983
**Sleep**	5.31 ± 3.46	4.92 ± 3.38	5.58 ± 3.50	.200
**Energy**	5.70 ± 3.33	5.45 ± 3.12	5.88 ± 3.47	.321
**Emotions**	5.41 ± 3.49	5.47 ± 3.12	5.37 ± 3.52	.770
**Body image**	4.08 ± 3.25	4.04 ± 3.12	4.11 ± 3.35	.982
**Sexual functions**	2.46 ± 2.43	2.42 ± 2.25	2.49 ± 2.55	.775
**Joint pain**	6.06 ± 3.32	6.01 ± 3.42	6.09 ± 3.26	.863

Note: Data are presented as mean ± SD. *P*-values represent comparisons between Crohn’s disease and ulcerative colitis using the Mann–Whitney *U* test.

### Risk factors associated with IBD-related disability

In univariate analysis (Table 3), education level (*P* = .026), azathioprine use (*P* = .019), any micronutrient deficiency (*P* = .007), self-reported vitamin D deficiency in the preceding 3 months (*P* = .011), and iron deficiency (*P* = .004) were associated with disability status. Azathioprine use was associated with a lower proportion of high disability, whereas any micronutrient deficiency, self-reported vitamin D deficiency in the preceding 3 months, and iron deficiency were associated with a higher proportion of high disability. All other evaluated sociodemographic, clinical, lifestyle, and treatment-related variables were not significantly associated with disability status in univariate analysis.

**Table 3:** Univariate analysis of factors associated with IBD-related disability.

Variable		Lower disability burden (IBD Disk score ≤40), *n* = 75	High disability (IBD Disk >40), *n* = 104	*P*-value
**Age category**	**18 and younger**	3 (4.0)	6 (5.8)	.061^a^
	**19-30**	31 (41.3)	40 (38.5)	
	**31-50**	27 (36.0)	51 (49.0)	
	**51 and older**	14 (18.7)	7 (6.7)	
**Gender**	**Male**	40 (53.3)	53 (51.0)	.754^a^
	**Female**	35 (46.7)	51 (49.0)	
**Marital status**	**Single**	26 (34.7)	40 (38.5)	.391^b^
	**Married**	47 (62.7)	64 (61.5)	
	**Divorced**	1 (1.3)	0 (0.0)	
	**Widow**	1 (1.3)	0 (0.0)	
**Education**	**Primary school**	5 (6.7)	3 (2.9)	**.026^b^**
	**High school**	17 (22.7)	44 (42.3)	
	**University**	48 (64.0)	54 (51.9)	
	**Postgraduate**	5 (6.7)	3 (2.9)	
**Disease type**	**Crohn’s disease**	43 (57.3)	63 (60.6)	.663^a^
	**Ulcerative colitis**	32 (42.7)	41 (39.4)	
**Disease duration**	**Less than 5 years**	21 (28.0)	32 (30.8)	.922^a^
	**5-10 years**	35 (46.7)	47 (45.2)	
	**More than 10 years**	19 (25.3)	25 (24.0)	
**Committed to treatment**	**No**	7 (9.3)	15 (14.4)	.306^a^
	**Yes**	68 (90.7)	89 (85.6)	
**Committed to regular follow-ups**	**No**	16 (21.3)	36 (34.6)	.053^a^
	**Yes**	59 (78.7)	68 (65.4)	
**Biologic treatment**	**No**	53 (70.7)	78 (75.0)	.518^a^
	**Yes**	22 (29.3)	26 (25.0)	
**5-aminosalicylates**	**No**	48 (64.0)	73 (70.2)	.382^a^
	**Yes**	27 (36.0)	31 (29.8)	
**Systemic steroids**	**No**	70 (93.3)	90 (86.5)	.145^a^
	**Yes**	5 (6.7)	14 (13.5)	
**Azathioprine**	**No**	39 (52.0)	72 (69.2)	**.019^a^**
	**Yes**	36 (48.0)	32 (30.8)	
**Other medications**	**No**	64 (85.3)	81 (77.9)	.210^a^
	**Yes**	11 (14.7)	23 (22.1)	
**Hospitalization**	**No**	31 (41.3)	33 (31.7)	.126^a^
	**Yes**	44 (58.7)	71 (68.3)	
**Had surgery**	**No**	62 (82.7)	86 (82.7)	.996^a^
	**Yes**	13 (17.3)	18 (17.3)	
**Type of surgery** ^†^	**Bowel resection**	10 (90.9)	9 (56.3)	.143^b^
	**Hemorrhoid/Fistula**	0 (0.0)	5 (31.3)	
	**Other abdominal surgery (appendix, cholecystectomy)**	1 (9.1)	2 (12.5)	
**BMI**	**Underweight**	3 (4.0)	9 (8.7)	.357^a^
	**Normal**	35 (46.7)	53 (51.0)	
	**Overweight**	19 (25.3)	27 (26.0)	
	**Obese**	17 (22.7)	15 (14.4)	
**Cigarette smoker**	**No**	51 (68.0)	62 (59.6)	.251^a^
	**Yes**	24 (32.0)	42 (40.4)	
**Smoker (other than cigarettes)**	**Nothing**	63 (84.0)	86 (82.7)	.086^b^
	**Waterpipe**	9 (12.0)	18 (17.3)	
	**Vape**	3 (4.0)	0 (0.0)	
**Diabetes mellitus**	**No**	70 (93.3)	102 (98.1)	.132^b^
	**Yes**	5 (6.7)	2 (1.9)	
**Hypertension**	No	68 (90.7)	101 (97.1)	.097^b^
	Yes	7 (9.3)	3 (2.9)	
**Dyslipidemia**	No	73 (97.3)	101 (97.1)	1.000^b^
	Yes	2 (2.7)	3 (2.9)	
**Behçet’s disease**	No	74 (98.7)	103 (99.0)	1.000^b^
	Yes	1 (1.3)	1 (1.0)	
**Rheumatoid arthritis**	No	73 (97.3)	98 (94.2)	.471^b^
	Yes	2 (2.7)	6 (5.8)	
**Psoriasis**	No	72 (96.0)	104 (100)	.072^b^
	Yes	3 (4.0)	0 (0)	
**Osteoporosis**	No	74 (98.7)	103 (99.0)	1.000^b^
	Yes	1 (1.3)	1 (1.0)	
**Any deficiencies**	No	54 (72.0)	54 (51.9)	**.007^a^**
	Yes	21 (28.0)	50 (48.1)	
**Calcium deficiency**	No	72 (96.0)	100 (96.2)	1.000^b^
	Yes	3 (4.0)	4 (3.8)	
**B12 deficiency**	No	70 (93.3)	89 (85.6)	.104^a^
	Yes	5 (6.7)	15 (14.4)	
**Vitamin D deficiency**	No	68 (90.7)	79 (76.0)	**.011^a^**
	Yes	7 (9.3)	25 (24.0)	
**Iron deficiency**	No	64 (85.3)	69 (66.3)	**.004^a^**
	Yes	11 (14.7)	35 (33.7)	

Note: Data are presented as *n* (%) within disability category unless otherwise indicated. Micronutrient deficiencies were self-reported and referred to the preceding 3 months. Values in bold indicate statistically significant associations (P < 0.05).

a Pearson’s chi-square test.

b Fisher’s exact test.

† Type-of-surgery percentages are calculated among participants with available surgery subtype data within each disability category (lower disability burden: *n* = 11; high disability: *n* = 16).

### Independent predictors of IBD-related disability

In the multivariable binary logistic regression model (Table 4), analysis showed that self-reported vitamin D deficiency in the preceding 3 months remained independently associated with higher odds of high IBD-related disability (aOR 2.93; 95% CI 1.07–8.06; *P* = .037). In contrast, azathioprine use was independently associated with lower odds of high IBD-related disability (aOR 0.421, 95% CI 0.213-0.829, *P* = .012). No other sociodemographic, clinical, lifestyle, or treatment-related variables included in the model were significantly associated with disability after adjustment.

**Table 4:** Multivariable logistic regression analysis of factors associated with high IBD-related disability.

Variable	Category	**aOR**	95% CI	***P*-value**
**Age category**	18 and younger	Reference		
	19-30	0.677	0.134-3.429	.637
	31-50	0.937	0.185-4.748	.938
	51 and older	0.213	0.034-1.327	.097
**Education**	Primary school	Reference		
	High school	2.949	0.569-15.277	.198
	University	1.210	0.243-6.016	.816
	Postgraduate	0.983	0.102-9.446	.988
**Vitamin D deficiency**	No	Reference		
	Yes	2.932	1.066-8.060	**.037**
**Azathioprine**	No	Reference		
	Yes	0.421	0.213-0.829	**.012**
**Gender**	Male	Reference		
	Female	1.038	0.497-2.168	.922
**Smoker**	No	Reference		
	Yes	0.991	0.454-2.165	.983
**Iron deficiency**	No	Reference		
	Yes	2.056	0.867-4.878	.102

Note: Reference category shown for each variable. Vitamin D and iron deficiencies were self-reported and referred to the preceding 3 months. Values in bold indicate statistically significant associations (P < 0.05).

Abbreviations: aOR, adjusted odds ratio; CI, confidence interval.

### Correlation analysis

A Spearman’s correlation analysis (Table 5) was performed to assess the relationship between IBD-related disability scores and continuous variables. Among patients with CD, the number of cigarettes smoked per day showed a weak but statistically significant positive correlation with IBD Disk scores (*ρ* = 0.229; *P* = .018). There were no significant correlations between IBD-related disability and age, disease duration, BMI, or years of marriage.

**Table 5:** Spearman correlation of total IBD Disk score with selected continuous variables overall and by disease type.

Variable	Overall, *ρ*	*P*-value	CD, *ρ*	*P*-value	UC, *ρ*	*P*-value
**Age**	−0.026	.733	0.000	.999	−0.060	.615
**Disease duration (years)**	−0.082	.275	−0.185	.058	0.064	.592
**BMI**	−0.043	.568	−0.096	.331	0.085	.474
**Cigarette number per day**	0.112	.134	**0.229**	**.018**	−0.113	.340
**Years of marriage**	−0.093	.330	−0.034	.810	−0.100	.455

Note: Analyses were performed using available data within each group. Values in bold indicate statistically significant associations (P < 0.05).

Abbreviation: ρ, Spearman’s rank correlation coefficient.

### Exploratory subgroup analysis

Exploratory subgroup analysis was performed according to biologic therapy status and prior surgical history. Mean total IBD Disk scores were similar between patients receiving biologic therapy and those not receiving biologic therapy (44.15 ± 22.86 vs 45.74 ± 23.40, *P* = .665). Likewise, mean total IBD Disk scores were similar between patients with prior surgical history and those without prior surgery (47.45 ± 22.74 vs 44.86 ± 23.35, *P* = .512). No significant differences were observed across individual IBD Disk domain scores in either comparison (Table S1).

## Discussion

This cross-sectional study assessed disability among Palestinian patients with IBD using the IBD Disk and found that more than half of the cohort experienced high levels of disability. The most impaired domains observed were joint pain, tiredness, emotional difficulties, and sleep disturbance. Cigarettes smoked per day showed a weak positive correlation with IBD Disk scores among patients with CD. In adjusted analysis, self-reported vitamin D deficiency in the preceding 3 months was associated with higher odds of high disability, whereas azathioprine use was associated with lower odds. Given the cross-sectional design, reliance on self-reported clinical exposures, and absence of objective disease activity measures, these associations should be interpreted as associative rather than causal and may partly reflect unmeasured differences in underlying disease activity or severity.

The disability level observed in this study exceeds those reported in several regional and international studies suggesting a comparatively greater functional burden. A Western study measuring IBD-related disability reported a mean score of 39.3 (±23.0), while another Saudi-based study reported a mean total IBD score of 20.70 (±18.69) and a 19% prevalence of IBD-related disability. Disability levels in this study were similar between CD and UC patients, mirroring findings in the Saudi-based and Western studies showing comparable disability levels. This suggests that disease type isn’t strictly reflective of disability and functional impairment. Higher scores in the joint pain domain were also reported in our study compared to those in the Western and Saudi Arabian studies, while the sexual function domain was consistently reported as the lowest across the studies.^22^^,^^23^ These differences may reflect variations in disease perception, access to specialized care, treatment pathways, and burden of extraintestinal manifestations. They may also reflect unmeasured clinical heterogeneity, including disease extent, which was not available in our dataset. The higher joint pain scores observed in our cohort may reflect an underrecognized burden of musculoskeletal extraintestinal manifestations and possible gaps in rheumatologic assessment and follow-up among patients with IBD. Since extraintestinal manifestations were not comprehensively characterized in our dataset, their specific contribution to the overall disability profile could not be fully determined. The sexual function domain being consistently reported as the lowest domain in literature is likely influenced by cultural norms and stigma surrounding the topic resulting in reluctance of patients to disclose sensitive information.

Azathioprine use in this study was associated with a lower burden of IBD-related disability. This observation aligns with previous work showing that thiopurines can support sustained remission and enhance patient-reported quality of life in appropriately selected individuals.^24^ A recent cohort noted that patients maintained on azathioprine often report better health-related quality of life outcomes compared with those managed with alternative therapies.^25^ However, this relationship must be interpreted cautiously. In clinical practice, patients who continue azathioprine typically represent a group with relatively stable or less aggressive disease, whereas escalation to biologic therapy usually occurs in the setting of more severe, refractory, or complicated IBD.^26–28^ This therapeutic “channeling” may partly explain the lower disability scores observed in azathioprine users, reflecting milder underlying disease rather than a direct comparative advantage of thiopurines over biologics. Even so, the observed association may reflect the better day-to-day functioning seen among patients whose disease is adequately controlled on a well-tolerated immunomodulator, although this interpretation remains speculative in the absence of objective disease activity measures.

The proportion of patients receiving biologic therapy in our cohort was relatively low (26.8%). While the relatively low proportion of patients receiving biologic therapy in our cohort may reflect a milder disease profile in some patients, it may also be related to local prescribing patterns, delayed treatment escalation, or limitations in treatment access. However, the present study was not designed to distinguish between these possibilities and detailed data on duration of treatment, prior treatment escalation, and combination therapy were not available, which further limits interpretation of this finding. Additionally, exploratory subgroup analyses did not demonstrate significant differences in total or domain-specific IBD Disk scores according to biologic therapy status. However, this finding should be interpreted cautiously, as biologic use alone may not adequately reflect disease severity, particularly in the absence of data on disease extent and activity.

Self-reported vitamin D deficiency in the preceding 3 months was independently associated with higher odds of high IBD-related disability in our analysis. This finding is consistent with previous literature linking low vitamin D status with higher disease activity and poorer health-related quality of life in IBD.^29^^,^^30^ Experimental and clinical data indicate that vitamin D supports mucosal healing through antimicrobial activity, modulation of intestinal inflammation, and enhancement of epithelial barrier integrity, providing a biological rationale for its potential contribution to disease control.^31^ Moreover, a recent meta-analysis of randomized trials reported that vitamin D supplementation as an adjuvant significantly reduced the rate of relapse in IBD by 64%, reinforcing the potential clinical relevance of maintaining adequate levels.^32^ Vitamin D deficiency in IBD patients has been linked to several clinical risk factors, including intestinal resection, stricture complications, early corticosteroid requirement, and extensive colonic involvement in UC.^33^ However, this association should be interpreted cautiously because vitamin D deficiency was self-reported rather than laboratory confirmed, which introduces the possibility of misclassification bias. The questionnaire captured self-reported vitamin D deficiency within the preceding 3 months, and current supplement and medication use was recorded separately. However, no data were available regarding laboratory values, deficiency severity, or whether the reported deficiency represented a prior diagnosis, a persistent deficiency, or a treated deficiency. It is therefore not possible to determine whether self-reported recent vitamin D deficiency itself was associated with greater disability, or whether it served as a marker of more active disease, poorer general health, extraintestinal burden, or other unmeasured factors. This distinction is particularly important because objective disease activity measures were not available in the present study. Future studies incorporating objective laboratory and disease activity data are needed to better clarify this relationship.

The weak positive correlation observed between cigarette number per day and IBD-related disability among patients with CD is in line with prior evidence. One study demonstrated that smoking not only contributes to the development of CD, but is also associated with poorer prognosis, reduced treatment response, and increased complications.^34^ Another study encourages the immediate cessation of smoking in CD patients, due to the worse outcomes smoking CD patients had, even with treatment, whether using biologics or surgery.^35^ However, in our study, smoking was only weakly correlated with disability scores, and no objective disease activity measures were available; therefore, the relationship should be interpreted cautiously.

### Strengths and limitations

This study has several strengths. It is the first study in Palestine that addresses and measures disability in IBD patients using the IBD-Disk, providing valuable insight from an understudied population. The sample size obtained, exceeding the minimum required sample size, enhances the robustness of the analysis. Additionally, the Arabic version of the IBD Disk used in this study underwent a structured translation and back-translation process, and demonstrated strong internal consistency (Cronbach’s *α* = 0.89), supporting its reliability for use in this context. Despite these strengths, this study has several limitations. First, the cross-sectional design does not allow causal inference, and the observed relationships should be interpreted as associations instead. Second, the study relied largely on self-reported data, including treatment history, comorbidities, smoking exposure, hospitalization, surgery, and micronutrient deficiencies, which introduces potential recall and misclassification bias. In particular, vitamin D deficiency was assessed by self-report for the preceding 3 months rather than by laboratory confirmation, and no data were available regarding laboratory values, deficiency severity, or whether the reported deficiency represented prior, persistent, or treated deficiency. Third, we did not collect objective measures of disease activity such as clinical indices, inflammatory biomarkers, or endoscopic assessments. Accordingly, it was not possible to determine whether the observed disability primarily reflected active intestinal disease, extraintestinal manifestations, psychosocial factors, or a combination of these influences. Because disease activity is strongly associated with disability in IBD, the absence of objective activity data limits interpretation of the observed associations. Fourth, several potentially important predictors of disability were not systematically assessed or comprehensively characterized, including extraintestinal manifestations, depression, anxiety, socioeconomic status, and healthcare-access factors. These variables may influence disability independently of intestinal disease activity and may have contributed to the burden observed in this cohort. Fifth, we did not collect detailed disease-phenotype data such as disease extent or distribution, which may influence disability burden and could have differed between patient groups. Sixth, detailed treatment characterization, including duration of biologic exposure and combination therapy, was not available. We were also unable to determine whether the relatively low biologic-use rate reflected disease severity, access limitations, or local treatment practices. Finally, participants were recruited from general outpatient gastroenterology clinics in the Northern West Bank using convenience sampling, which may have introduced selection bias. Although these were general clinics rather than specialized IBD referral clinics, the sample may still overrepresent patients who are more engaged with follow-up care and underrepresent those who do not attend regularly. As a result, the severity profile and disability burden observed in this study may not fully reflect all patients with IBD in the community. This also limits the generalizability of the findings to patients outside this clinic-attending population and to other regions.

### Implications and recommendations

The findings of this study highlight the importance of integrating disability assessment into routine IBD care. Using the IBD Disk offers a rapid evaluation targeting several dimensions. This can help physicians identify patients with substantial functional burden regardless of traditional markers which may appear stable. The IBD Disk may help identify patients reporting substantial functional burden in routine care. Future longitudinal studies should incorporate objective disease activity measures, disease extent, extraintestinal manifestations, depression and anxiety, healthcare-access factors, and detailed biologic exposure data, including exposure duration, to better define independent predictors of disability.

## Conclusion

This study revealed that the prevalence of IBD disability in Palestine was 58.1%, with joint pain being the domain with the highest scores. This highlights the substantial disability burden observed among Palestinian patients with IBD. Azathioprine use was associated with lower disability scores, whereas self-reported vitamin D deficiency in the preceding 3 months was associated with higher disability. In addition, cigarettes smoked per day showed a weak positive correlation with IBD Disk scores among patients with CD, whereas smoking status was not independently associated with high disability in the multivariable model. These findings should be interpreted cautiously given the cross-sectional design, self-reported exposures, and absence of objective disease activity measures. All in all, implementing routine disability assessment using the IBD Disk may support early identification of high-risk patients and guide more targeted clinical interventions to improve outcomes for individuals living with IBD.

## Supplementary Material

otag079_Supplementary_Data

## Data Availability

The data and materials used in this work are available from the corresponding author upon request.
