# Supplementary material for: Disability among Palestinian patients with inflammatory bowel disease: evaluation using the IBD Disk and identification of associated factors
Source: Crohns Colitis 360. 2026 Jul 17;8(3):otag079. doi: 10.1093/crocol/otag079 (PMC13418206; doi:10.1093/crocol/otag079)
Supplement: otag079_Supplementary_Data [file otag079_supplementary_data.docx]

**Supplementary Table S1. Exploratory comparison of total and domain-specific IBD Disk scores according to biologic therapy status and prior surgical history**

| **A. Biologic therapy status** |  |  |  |
| --- | --- | --- | --- |
| **Variable** | **No biologic**  **(n = 131),**  **mean ± SD** | **Biologic**  **(n = 48), mean ± SD** | **p-value** |
| **Total IBD Disk score** | 45.74±23.40 | 44.15 ± 22.86 | 0.665 |
| **Abdominal pain** | 4.98 ±3.21 | 5.02 ± 3.03 | 0.959 |
| **Regulating defecation** | 3.60 ± 3.13 | 3.40 ± 2.86 | 0.876 |
| **Interpersonal interactions** | 3.48 ± 3.19 | 3.77 ± 3.14 | 0.472 |
| **Education and work** | 4.19 ± 3.42 | 4.17 ± 3.49 | 0.959 |
| **Sleep** | 5.42 ± 3.52 | 5.02 ± 3.30 | 0.490 |
| **Energy** | 5.92 ± 3.31 | 5.13 ± 3.33 | 0.143 |
| **Emotions** | 5.43 ± 3.57 | 5.35 ± 3.27 | 0.900 |
| **Body image** | 4.27 ± 3.29 | 3.56 ± 3.10 | 0.146 |
| **Sexual functions** | 2.46 ± 2.49 | 2.48 ± 2.26 | 0.709 |
| **Joint pain** | 5.99 ± 3.42 | 6.25 ± 3.05 | 0.763 |
| **B. Prior surgical history** |  |  |  |
| **Variable** | **No surgery**  **(n = 148),**  **mean ± SD** | **Prior surgery**  **(n = 31), mean ± SD** | **p-value** |
| **Total IBD Disk score** | 44.86 ± 23.35 | 47.45 ± 22.74 | 0.512 |
| **Abdominal pain** | 5.09 ± 3.19 | 4.52 ± 3.01 | 0.405 |
| **Regulating defecation** | 3.54 ± 3.03 | 3.58 ± 3.21 | 0.989 |
| **Interpersonal interactions** | 3.52 ± 3.15 | 3.74 ± 3.31 | 0.652 |
| **Education and work** | 4.16 ± 3.44 | 4.32 ± 3.41 | 0.618 |
| **Sleep** | 5.18 ± 3.47 | 5.94 ± 3.36 | 0.272 |
| **Energy** | 5.65 ± 3.33 | 5.97 ± 3.33 | 0.653 |
| **Emotions** | 5.35 ± 3.48 | 5.68 ± 3.58 | 0.618 |
| **Body image** | 3.92 ± 3.22 | 4.87 ± 3.30 | 0.107 |
| **Sexual functions** | 2.47 ± 2.50 | 2.42 ± 2.09 | 0.717 |
| **Joint pain** | 5.99 ± 3.32 | 6.42 ± 3.34 | 0.403 |

Data are presented as mean ± SD. P-values represent comparisons between subgroups using the Mann–Whitney U test.
